# *OsARP6* Is Involved in Internode Elongation by Regulating Cell-Cycle-Related Genes

**DOI:** 10.3390/biom11081100

**Published:** 2021-07-26

**Authors:** Aziz Ul Ikram, Yong Ding, Yanhua Su

**Affiliations:** Ministry of Education Key Laboratory for Membraneless Organelles and Cellular Dynamics, Hefei National Laboratory for Physical Sciences at the Microscale, Division of Molecular Cell Biophysics, Chinese Academy of Sciences (CAS) Center for Excellence in Molecular Plant Sciences, School of Life Sciences, Division of Life Sciences and Medicine, University of Science and Technology of China, Hefei 230027, China

**Keywords:** *OsARP6*, SWR1-C, H2A.Z, internode elongation, rice

## Abstract

The SWR1 complex (SWR1-C) is important for the deposition of histone variant H2A.Z into chromatin to regulate gene expression. Characterization of SWR1-C subunits in *Arabidopsis thaliana* has revealed their role in variety of developmental processes. *Oryza sativa* actin related protein 6 (OsARP6) is a subunit of rice SWR1-C. Its role in rice plant development is unknown. Here, we examined the subcellular localization, expression patterns, and loss of function phenotypes for this protein and found that OsARP6 is a nuclear localized protein, and is broadly expressed. OsARP6 interacted with OsPIE1, a central ATPase subunit of rice SWR1-C. The *osarp6* knockout mutants displayed pleiotropic phenotypic alterations in vegetative and reproductive traits, including semi-dwarf phenotype, lower tillers number, short leaf length, changes in spikelet morphology, and seed abortion. Microscopic thin sectioning of the top internode revealed that the dwarf phenotype of *osarp6* was due to reduced number of cells rather than reduced cell length. The altered transcript level of genes involved in cell division suggested that *OsARP6* affects cell cycle regulation. In addition, H2A.Z levels were reduced at the promoters and transcription start sites (TSS) of the regulated genes in *osarp6* plants. Together, these results suggest that OsARP6 is involved in rice plant development, and H2A.Z deposition.

## 1. Introduction

Plant height, determined by the length of internodes as well as the number of elongated internodes, is an important agronomic trait that affects yield potential directly [1]. Suitable height in rice and wheat, in combination with new fertilizer and pesticide technologies, doubled production in most parts of the world during “Green Revolution” [2]. Moreover, increased total biomass, resistance to lodging and tolerance to crowding result from their short stature [3,4]. Therefore, it is necessary to clarify the signal network regulating plant height.

In eukaryotic organisms, DNA is tightly wrapped inside the nucleus into a compact structure known as chromatin, which blocks transcription factors and other DNA binding proteins to access DNA targets and perform their function [5]. Histone modifications, ATP-dependent chromatin remodeling and replacement of canonical histones with specialized variants regulate chromatin structure to balance chromatin packaging and transcriptional access [6,7,8]. Except for H4, all canonical histones have known variants, with H2A having the largest number of variants [9]. Among H2A variants, H2A.Z is the most conserved and affects multiple biological processes, including regulation of gene expression [10,11].

The SWR1 complex (SWR1-C), a member of the Inositol Requiring 80 (INO80) family of remodelers, mediates deposition of H2A.Z into nucleosomes. SWR1-C was initially discovered in yeast (*Saccharomyces cerevisiae*) [12]. Only five of the 14 proteins in the yeast SWR1-C are essential for viability; the remainder, including the SWR1 ATPase, enhance complex function [12]. Most of the SWR1-C components are conserved from yeast to *Arabidopsis* [9,13,14]. *Arabidopsis PIE1*, encoding the ATPase domain containing chromatin-remodeling factor, directly binds to H2A.Z variants, including HTA8, HTA9, and HTA11 [15]. Loss of *PIE1* leads to early flowering through a reduction in the expression of *FLOWERING LOCUS C (FLC)*, reduced H2A.Z deposition, and pleiotropic developmental abnormalities including short siliques and fertility defects [16,17,18]. Silencing of *AtARP4* results in altered organization of plant organs, early flowering, delayed flower senescence and high levels of sterility [19]. *SWC6* loss-of-function mutants also display pleiotropic phenotypes characterized by serrated leaves, frequent absence of inflorescence internodes, and flowers with an abnormal number and size of organs, as well as early flowering due to reduced *FLC* expression [18,20]. *SWC4 RNAi (swc4i)* and *yaf9a yaf9b* double mutant plants display pleiotropic phenotypic defects in both vegetative and reproductive development, including early senescence and chlorotic leaves, abnormal flowering, and reduced plant and organ size [21,22]. *SWC4* represses transcription of a number of genes, including the floral integrator *FT* and key transcription factors, mainly by modulating H2A.Z deposition [22]. ACTIN-RELATED PROTEIN6 (ARP6), one of the nonessential subunits of the yeast SWR1-C, is required for normal growth of yeast. In the yeast SWR1-C, ARP6 facilitates binding between other subunits, such as Swc2, and the ATPase domain of SWR1. In *Arabidopsis*, ARP6 acts in the nucleus [23,24] to modulate gene expression in vegetative development and repression of flowering. The early flowering of *arp6* mutants is associated with reduced expression of the central floral repressor gene *FLC* as well as *MADS AFFECTING FLOWERING 4 (MAF4)* and *MAF5* [23]. In rice, genome-wide H2A.Z distribution was altered in *OsARP6* RNAi plants [25]. The H2A.Z deposition mediated by *OsARP6* in gene bodies largely resulted in downregulation, whereas H2A.Z at the transcription start sites (TSS) was positively correlated with the expression of some genes with housekeeping functions [25].

The SWR1-C is a likely candidate to regulate gene expression during cell division. *Arabidopsis* SWR1-C components mutants such as ARP4, ARP6, SWC4 and YAF9 results in dwarfed plant morphology, decreased leaf size and number, and reduced silique size [19,21,22,23]. Microscopic analysis investigating the smaller size of *yaf9a yaf9b* double mutants and of knock-down plants for *SWC4* encoding an interactor of YAF9A, found a shared role for these proteins in the regulation of leaf cell proliferation and expansion [21,22]. Similarly, smaller leaves from *arp6-1* plants are caused by fewer total cells, rather than a normal number of smaller cells [23]. Together, all these observations suggest a potential role of SWR1-C in regulating cell cycle [17,21,22].

Although SWR1-C is involved in plant development, environmental response, and transcription regulation in *Arabidopsis*, the functions of SWR1-C in rice development remain unclear. Here, we report that *Oryza staiva* ARP6 (OsARP6), a subunit of rice SWR1-C, participates in rice plant vegetative and reproductive development. We found that *OsARP6* is broadly expressed in the whole plant, with high expression levels in panicle and leaves at the heading stage. Loss of *OsARP6* function in rice leads to dwarf phenotype with defects in cell proliferation. The transcription levels of cell-cycle genes were down regulated in *osarp6* mutants. The CHIP-qPCR results showed that down regulated transcription in *osarp6* plants was due to reduced H2A.Z levels at the promoters and TSS of the cell-cycle-related genes. Taken together, our results are consistent with the role of *OsARP6* in the chromatin-level control of multiple genes.

## 2. Materials and Methods

The plants used in the study are in the *Oryza sativa* ssp. *japonica* cv Nipponbare background. The *osarp6-1* and *osarp6-2*, were generated by CRISPR/Cas9. All rice plants were grown in Hefei (Anhui, China), and Lingshui (Hainan, China) in 2018, 2019 and 2020. For mutants generated by CRISPR/Cas9, the oligonucleotides used for targeted mutagenesis were designed with the help of the CRISPR-P and CRISPR-PLANT tools [26,27] and are listed in Table S2. The oligonucleotides were inserted into the CRISPR/Cas9 vector pHUN4c12 with BsaI. The binary constructs were then introduced into the *Agrobacterium tumefaciens* strain *EHA105*. Embryonic calli from mature rice seeds were transformed by co-cultivation, selected with 50 mg/L hygromycin, and used to regenerate transgenic plants.

### 2.1. Subcellular Localization

A *35S:OsARP6-GFP* fusion vector was constructed by cloning the full-length *OsARP6* coding sequence (CDS) without a stop codon in the *pUC19-eGFP* vector. *35S:GFP* was used as a control. The inserted gene was confirmed by gene sequencing followed by transient expression in rice protoplast. Rice protoplast isolation and transformation were performed as described previously [28]. Briefly, the stem and sheath tissues of 7–10 d-old rice seedlings were cut into 0.5 mm strips and immediately transferred into 0.6 M mannitol. After enzymatic digestion for 8 h, an equal volume of W5 solution (154 mM NaCl, 125 mM CaCl_2_, 5 mM KCl and 2 mM MES at pH 5.7) was added to the sample, followed by resuspension in MMG solution (0.4 M mannitol, 15 mM MgCl_2_ and 4 mM MES, pH 5.7). After 16 h incubation in the dark at 26 °C, the green fluorescence of *35S:GFP* and *35S:OsARP6-GFP* in protoplast was visualized using a Zeiss LSM 880 (Jena, Germany) confocal laser scanning microscope.

### 2.2. Yeast Two-Hybrid Assay (Y2H)

The yeast two-hybrid assay (Y2H) was performed according to the manufacturer’s protocol (Clontech, Mountain View, CA, USA, user’s manual 630489). Briefly, the *Saccharomyces cerevisiae* strain *AH109* was co-transformed with the bait and prey constructs, in *pGBKT7* and *pGADT7* respectively. The yeast was scored for protein interaction based on its ability to grow on synthetic defined medium lacking tryptophan (Trp), leucine (Leu), histidine (His) and adenine (Ade). The primers used to generate the constructs are shown in Table S2.

### 2.3. Bimolecular Fluorescence Complementation (BiFC)

For bimolecular fluorescence complementation (BiFC), *OsPIE1* was cloned in *pUC-SPYNE* vector while *OsARP6* was cloned into the *pUC-SPYCE* vector. The rice protoplasts were co-transformed with the corresponding constructs and examined under a confocal laser scanning microscope (Zeiss LSM880, Jena, Germany). The primers used to generate the constructs are shown in Table S2.

### 2.4. Genotyping

To confirm the Cas9 mutants, primers were designed flanking the target regions. DNA was extracted from the mutants, and the target region was amplified and sequenced to check the desired mutation. The CRISPR/Cas9 constructs were segregated out in self-pollinated lines. T2 plants were used for phenotypic evaluation. The primers used for genotyping are listed in Table S2.

### 2.5. Histological Analyses

For histological analysis, the uppermost internodes were collected from field-grown mature plants and fixed in 3.7% formaldehyde, 5% acetic acid, and 50% ethanol at 4 °C overnight. The samples were auto-dehydrated in a LEICA ASP200S tissue processor, embedded in paraffin (Leica Biosystems, Richmond, VA, USA), and sectioned to 8 µm thicknesses using a LEICA RM2255 rotary microtome. After dewaxing with xylene and hematoxylin-eosin staining, the sections were observed under a light microscope (Zeiss, Axioscope A1 polarized light microscope, Jena, Germany).

### 2.6. Reverse Transcription and Quantitative PCR (RT-qPCR)

Total RNA from the leaves of 10-d-old plants was isolated and was reverse transcribed with oligo (dT) primers and random primers. The relative expression levels of individual genes were measured with gene-specific primers. Quantitative PCR analysis was performed with the CFX real-time PCR instrument (Bio-Rad, Hercules, CA, USA) and SYBR Green mixture (ChamQ, Vazyme Biotech Co., Ltd., Nanjing, China). The relative expression of the genes was quantitated with the 2^−ddct^ Ct calculation, using *ACTIN1* as the reference housekeeping gene for the expression analyses. The primers used for qPCR are listed in Table S2.

### 2.7. ChIP Assay

ChIP assay was performed as described previously [28,29]. Briefly, 3 g of 3-week-old plant material was crosslinked in 1% formaldehyde for 10 min and then quenched with 0.125 M glycine. Then, 5 µg of the specific antibodies anti-H2A.Z (ab4174, lot: GR292900-1, Abcam, Cambridge, UK), or control IgG serum, were added to the pre-cleared supernatants for an overnight incubation at 4 °C. The protein–DNA complexes were heated at 65 °C for 8 h to reverse the formaldehyde crosslinking. The sample was then extracted with phenol/chloroform and the DNA was precipitated in ethanol and resuspended in water. The purified DNA was analyzed by qPCR with the gene-specific primers shown in Table S2.

## 3. Results

### 3.1. Phylogenetic Analysis and Subcellular Localization of OsARP6

**Figure 1:**
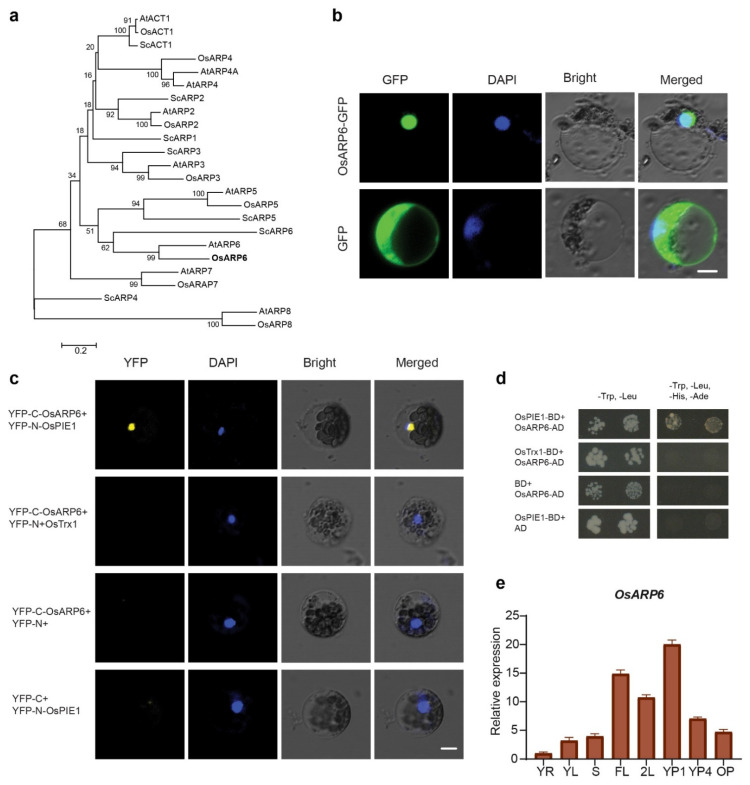
OsARP6 is nucleus localized and interacts with OsPIE1. (**a**) Phylogenetic analysis of actin-related proteins from yeast, *Arabidopsis* and rice. At, *Arabidopsis thaliana*; Os, *Oryza sativa*; Sc, *Saccharomyces cerevisiae*. OsARP6 is shown in bold. The analysis was performed by MEGA 5.2 using the neighbor-joining (NJ) method. (**b**) Subcellular localization of OsARP6. Confocal laser-scanning microscopy observations of rice protoplast expressing *35S:GFP* as a control or *35S:OsARP6-GFP* fusion protein. Scale bar = 10 µm. (**c**) OsARP6 fused to the C-terminus of yellow fluorescent protein (YFP) was tested for its ability to bind to N-terminus of YFP fused to OsPIE1 or the N-terminus of YFP fused to OsTrx1 (used as negative control). OsARP6 fused to C-terminus of YFP and OsPIE1 fused to N-terminus of YFP, were also tested for their ability to bind to N-terminus of YFP alone or C-terminus of YFP alone, respectively, added as negative control. Yellow fluorescence and a bright-field image were recorded, and the resulting images were merged. Twenty-five cells were examined for each transformation. Bar = 10 µm. (**d**) Yeast two-hybrid assay revealing the interaction between OsARP6 and OsPIE1. The growth of two dilutions (2 × 10^−2^ and 2 × 10^−3^) of yeast culture on synthetic defined (SD) medium lacking Trp, Leu, His and adenine is shown. (**e**) Expression pattern of *OsARP6* in different organs was determined by quantitative RT-qPCR and normalized against *ACTIN1*; YR, young root; YL, young leaf; S, shoot; YP1, young panicle stage 1; YP4, young panicle stage 4; OP, old panicle; FL, flag leaf at heading stage; 2L, second leaf to flag leaf at heading stage. Error bars indicate SD for three replicates.

*Oryza sativa* Actin-related protein 6-like (OsARP6) is a 429-amino-acid protein encoded by *LOC_Os01g16414*. Based on overall amino acid sequence similarity, OsARP6 is 63% and 25% identical to *Arabidopsis* and yeast ARP6 proteins, respectively. Phylogenetic analysis using the amino acid sequences of putative nuclear ARPs from rice, *Arabidopsis* and yeast were aligned along with conventional actins, and the resulting alignments were used to generate phylogenies, which indicated that OsARP6 does indeed belong to the ARP6 class, and thus is the conserved ortholog of *Arabidopsis* and yeast ARP6 proteins (Figure 1a). Multiple alignment analysis using conventional actins from human, yeast, *Arabidopsis* and rice as reference showed that OsARP6, along with *Arabidopsis* and yeast ARP6 proteins, contain Nucleotide-Binding Domain of the sugar kinase/HSP70/Actin super family (NBD_sugar-kinase_HSP70_actin) (Figure S1). Of the 17 key amino acid residues involved in nucleotide binding [30,31], 8, 9 and 6 are conserved in rice, *Arabidopsis* and yeast, respectively (Figure S1). To investigate the subcellular localization of OsARP6, we generated a *35S:OsARP6-GFP* vector and transiently expressed it in rice protoplast. We found that the OsARP6–GFP fusion protein was localized to the nucleus. As a control, free GFP was localized in both the cytosol and nucleus in protoplast (Figure 1b).

### 3.2. OsARP6 Interacts with OsPIE1

*Arabidopsis PHOTOPERIOD-INDEPENDENT EARLY FLOWERING1* (*PIE1*) encodes a putative DNA-dependent ATPase of the SNF2 family [15], closely related to yeast Swr1, human SRCAP, and Drosophila Domino proteins. *Arabidopsis* ARP6 interacts with PIE1 and SWC6 via the c-terminus [16]. To identify whether the OsARP6 is conserved from *Arabidopsis* to rice, we examined the interaction between OsARP6 and OsPIE1, a PIE1 ortholog of *Arabidopsis* in rice. The reconstituted YFP fluorescence was observed in the nucleus when YFP^C^-OsARP6 and YFP^N^-OsPIE1 were co-transformed into rice protoplast, whereas no fluorescence was observed when YFP^C^-OsARP6 was co-transformed with YFP^N^-OsTrx1, another nuclear protein (Figure 1c). The interaction of OsARP6 with OsPIE1 was also observed in yeast two hybrid (Y2H) analysis. We generated fusion proteins containing OsPIE1 with the binding domain (BD) and OsARP6 fused to activation domain (AD). The results revealed that OsARP6 bound strongly to OsPIE1, but not to OsTrx1, which was used as negative control (Figure 1d). Together, all these results suggest that the subunits of SWR1-C might be conserved in *Arabidopsis* and rice.

### 3.3. Expression Pattern of OsARP6

We examined the expression pattern of OsARP6 in different tissues by quantitative real-time PCR (RT-qPCR). The results showed that OsARP6 is broadly expressed in root, shoot, leaf and panicle, with much higher expression in panicle and leaf at the heading stage (Figure 1e). Expression analysis of OsARP6 using the Rice eFP Browser (http://www.bar.utoronto.ca/efprice/cgi-bin/efpWeb.cgi, access date 12 June 2021) showed similar results, indicating that OsARP6 was broadly expressed root, stem, leaf, panicle and seed, with much higher expression in somatic apical meristem (SAM), young panicle and mature leaf (Figure S2).

### 3.4. Generation of Rice osarp6 Mutants with CRISPR Cas9

**Figure 2:**
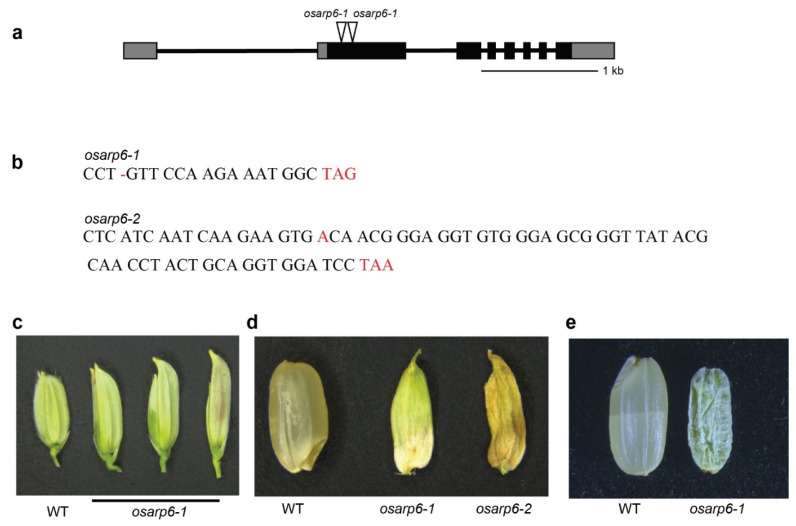
Gene structure of *OsARP6* and generation of *osarp6* mutants using CRISPR/Cas9. (**a**) Gene structure of *OsARP6*, showing exons (boxes), introns (lines), and Cas9 targets (triangles). (**b**) The deletion and insertion in *osarp6-1* and *osarp6-2* respectively and the stop codon caused by a shifted open reading frame (ORF) are shown in red. (**c**) Typical external appearance of wild-type and *osarp6* flowers. Spikelets just before anthesis are shown. (**d**) Typical seeds obtained from wild-type and homozygous *osarp6* plants. Seeds at 30 DAP are shown. (**e**) Comparison of wild-type seeds with homozygous seeds obtained from heterozygous *osarp6* plants. Seeds at 30 DAP are shown.

To investigate the function of *OsARP6*, we generated knockout *osarp6* mutants with the CRISPR/Cas9 system. Two 20-bp nucleotide sequences from the open reading frame in exon 1 of *OsARP6* were chosen as the target sites (Figure 2a). Sequencing results revealed that a single nucleotide deletion in the first exon in *osarp6-1* resulted in an early stop codon at the 45th amino acid in the Open Reading Frame (ORF), while a single nucleotide insertion in the first exon in *osarp6-2* resulted in an early stop codon at the 93rd amino acid in the ORF (Figure 2b). The sequencing results of the mutants are shown in Figure S3a,b. The homozygous knockout lines, *osarp6-1* and *osarp6-2*, failed to produce mature seeds. Therefore, we propagated the mutation through corresponding heterozygous lines. T2 generation of self-pollinated mutant plants was used for phenotype analysis.

### 3.5. Loss of OsARP6 Function Resulted in Pleotropic Phenotypes

The homozygous *osarp6* plants were sterile and could not develop mature seeds. The spikelet length of *osarp6* was longer than wild type (Figure 2c). The length of palea varied from normal to shorter, and was in some cases extremely reduced (Figure 2c). The seeds from homozygous *osarp6* mutants exhibited a filling problem, which could not be germinated (Figure 2d). However, examination of the seeds collected from heterozygous *osarp6* plants revealed that about 22% of seeds were severely affected (Figure 2e). The genotyping of such seeds confirmed that they carried homozygous loss of function mutation for *OsARP6*. The segregation analysis of heterozygous *osarp6* self-pollinated plants showed ratios of about 1:1.85:0.81 for WT, heterozygous *osarp6* and homozygous *osarp6* plants, which are close to the expected 1:2:1 ratio (*X*^2^ = 1.37 and *P* > 0.05) (Table 1). This indicates that additional gametophytic defects were not involved. Homozygous seeds obtained from heterozygous mutants could partially germinate and grow into mature plants.

**Figure 3:**
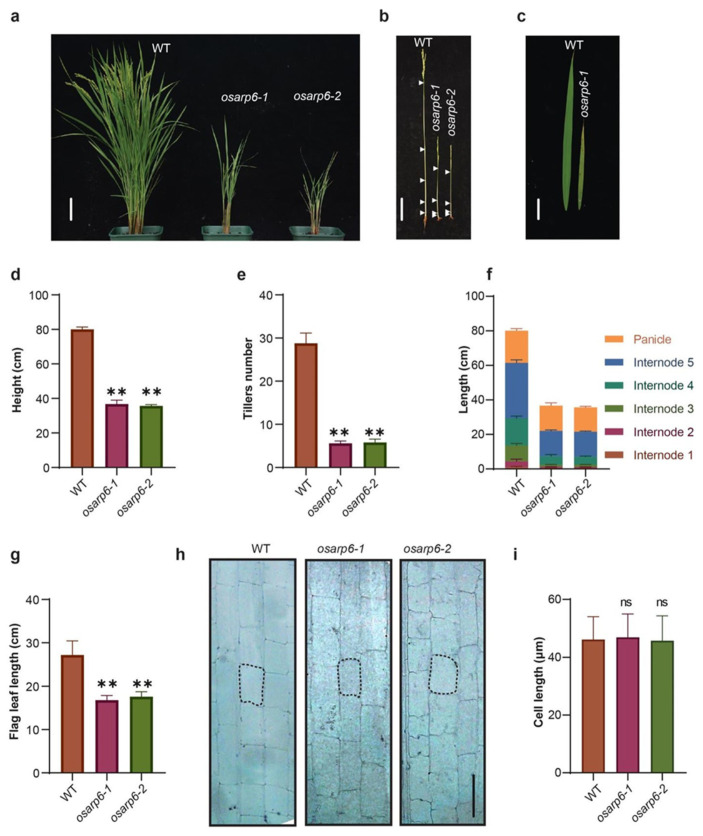
Phenotypes of homozygous *osarp6* plants. (**a**) Representative image of 120-day-old wild-type and *osarp6* homozygous plants. Scale bar = 10 cm. (**b**) Stems of the mature wild-type and homozygous *osarp6* plants. All leaves were removed to clearly show the elongated stems. White arrowheads indicate the nodes. Scale bar = 10 cm. (**c**) Leaf phenotype of 120-day-old wild-type and *osarp6* plants. Flag leaf, wild-type (left) and *osarp6* (right), are shown. Scale bar = 5 cm. (**d**) Quantification of height of wild-type and *osarp6* plants. Error bars indicate the SD (n = 12). Asterisks indicate *P* < 0.01 (**) as determined by Student’s *t* test analysis. (**e**) Quantification of tillers number from wild-type and *osarp6* plants. Error bars indicate the SD (n = 12). Asterisks indicate *P* < 0.01 (**) as determined by Student’s *t* test analysis. (**f**) Internode and panicle lengths of wild-type and *osarp6* plants. Error bars indicate the SD (n = 12). (**g**) Quantification of flag leaf length from wild-type and *osarp6* plants. Error bars indicate the SD (n = 10). Asterisks indicate *P* < 0.01 (**) as determined by Student’s *t* test analysis. (**h**) Longitudinal sections of the elongated zones of the 5th internode (uppermost internode) of wild-type and homozygous *osarp6* plants at the mature stage. Scale bar = 50 µm. (**i**) Quantification of cell length of the uppermost internode of wild-type and homozygous *osarp6* plants. Error bars indicate SD (n = 50). ns indicates non-significant as determined by Student’s *t* test analysis.

The homozygous knockout *osarp6* plants raised from the seeds obtained from self-pollinated heterozygous *osarp6* showed pleiotropic phenotypes, including the semi-dwarf phenotype, lower tillers number, and short leaf length (Figure 3a–g). Semi-dwarf phenotype and lower tillers number were also observed in heterozygous *osarp6* plants (Figure S4a–e), suggesting that *OsARP6* is crucial for rice development. Decrease in length was observed in all internodes with the top three internodes most affected (Table 2). Together, these results show that *OsARP6* is required for normal vegetative and reproductive growth of rice.

### 3.6. The Reduced Height of osarp6 Is Due to Defect in Cell Proliferation

To investigate whether dwarf morphology of *osarp6* plants was caused by defects in cell elongation and/or cell proliferation, the longitudinal cell morphology of the *osarp6* and WT internodes was observed. After the heading stage, the middle sections of the fifth internode (top) were collected, fixed, and sectioned, and the cell length was observed under microscope. The results showed that cell length was not affected in *osarp6* plants (Figure 3h,i). The ratios of cell number in a single row in 1 cm sections from top, middle and bottom portions in 5th internodes were not different in wild-type and *osarp6* mutants (Table S1). The estimated total number of cells in a single row in 5th internode in wild type was more than two times than *osarp6* mutants (Table S1). Together, these results showed that reduction in longitudinal cell number in the elongation zone may account for the short internodes in *osarp6* plants.

### 3.7. OsARP6 Is Involved Regulating the Expression of Various Cell-Cycle-Related Genes

**Figure 4:**
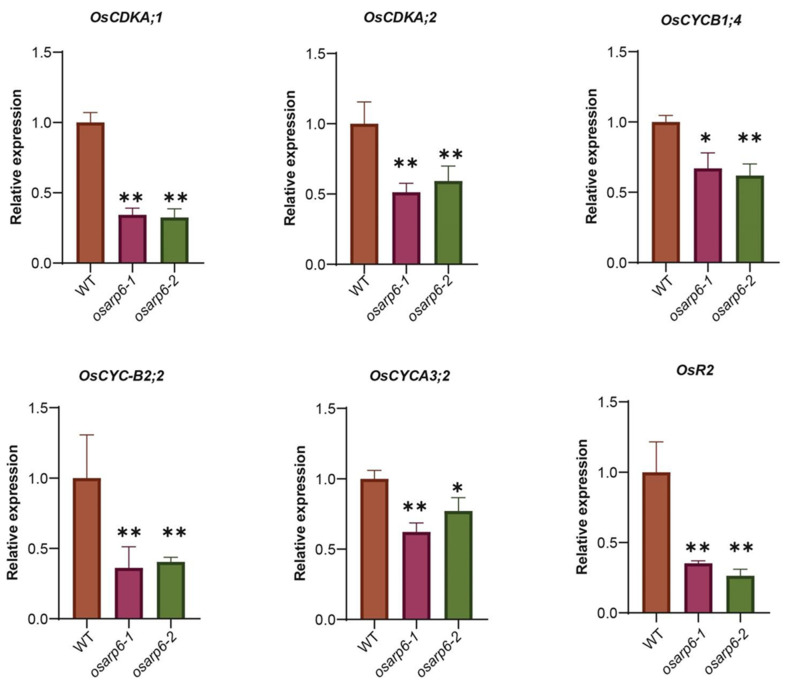
*OsARP6* promotes the expression of cell-cycle-regulatory genes. RNA was extracted from three-week-old seedlings, and RT-qPCR was performed using gene-specific primers. *ACTIN1* was used for internal control. Experiments were repeated at least three times, and the data from representative experiments shown are presented as means. Error bars indicate SD for three replicates. Asterisks indicate *P* < 0.05 (*) and *P* < 0.01 (**) compared with WT in the Student’s *t* test analysis.

The altered rate of cell proliferation in the *osarp6* mutant prompted us to examine the expression of cell-cycle-regulating genes. The cell division cycle is controlled in eukaryotes by cyclin-dependent kinases (CDKs) [32,33,34]. Total RNA was isolated from the leaves of 10-day-old seedlings of WT and *osarp6* mutant plants, and was subjected to RT-qPCR. Expression level of *OsCDKA2;1* and *CDKA;2*, involved in controlling both G1/S and G2/M transitions [35] were down regulated in *osarp6* mutants (Figure 4). Similarly, the expression level of the Cyclin-dependent kinase-activating kinase *R2*, which regulates S-phase progression [36], was greatly decreased in *osarp6* mutants compared to WT (Figure 4). The kinase activity of CDKs is dependent on the binding of positive regulators known as cyclins, first identified as proteins showing a cyclical pattern of accumulation and destruction during the synchronous divisions that characterize early embryonic development in marine invertebrates [37]. The A-type cyclins generally appear at the onset of S-phase [38,39,40,41]. In contrast, the B-type cyclins are induced in late S to G2 phase, reach their maximum levels during mitosis, and are eventually ubiquitously degraded prior to anaphase via the functions of the destruction boxes (D-box) located in their N-termini [42,43]. Expression analysis of different cyclins genes showed that *OsCYCA3;2*, *OsCYCB1;4*, and *OsCYCB2;2* were significantly downregulated in *osarp6* mutants (Figure 4). Therefore, our results indicate that the altered rate of cell proliferation in *osarp6* might be due to the altered expression level of cell cycle specific genes.

### 3.8. OsARP6 Is Required for H2A.Z Deposition at the Promoters and TSS of Cell-Cycle-Related Genes

**Figure 5:**
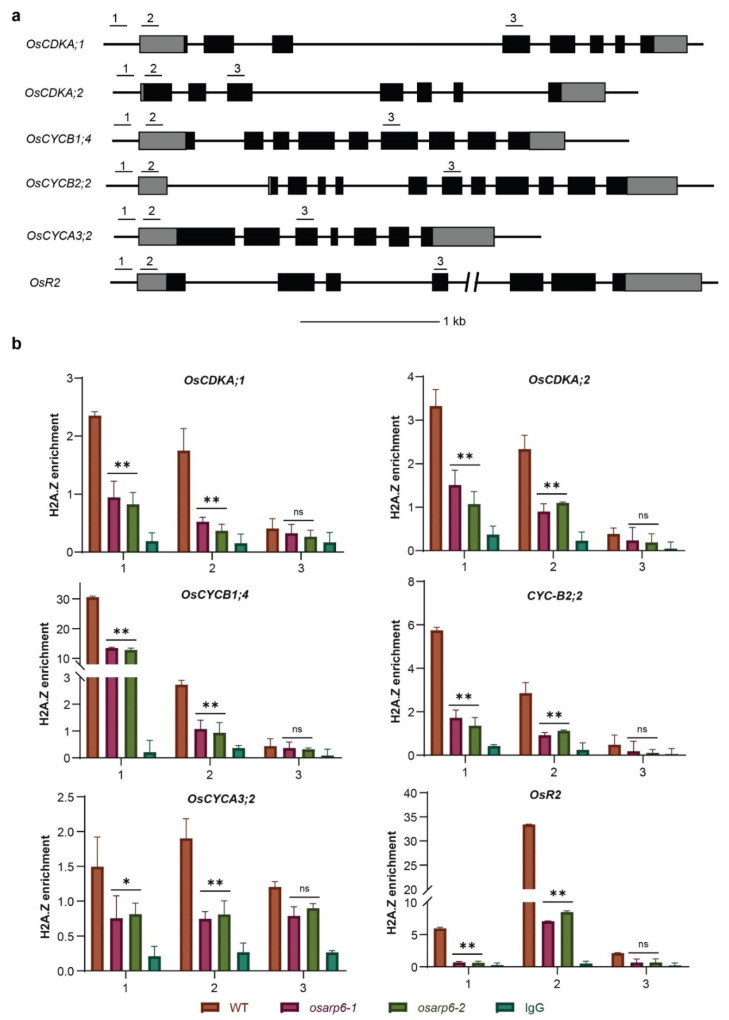
*OsARP6* is responsible for H2A.Z deposition at promoters and transcription start sites (TSS) of the cell-cycle genes. (**a**) Regions used for checking H2A.Z levels at cell-cycle-regulatory genes in wild-type and *osarp6* mutants. 1, 2 and 3 respectively denote promoter, TSS and gene body. (**b**) H2A.Z levels at cell-cycle-regulatory genes in wild-type and *osarp6* plants using H2A.Z specific antibodies. IgG was used as negative control. *ACTIN1* was used as internal control. Experiments were repeated at least three times, and the data from representative experiments shown are presented as means. Error bars indicate SD for three replicates. Asterisks indicate *P* < 0.05 (*) and *P* < 0.01 (**) and ns indicates non-significant compared with WT in the Student’s *t* test analysis.

Yeast and *Arabidopsis* ARP6 proteins, as part of the SWR1-C, are involved in H2A.Z deposition [17,21,44]. We speculated as to whether mutation in rice *OsARP6* may also cause lower deposition of H2A.Z at the cell-cycle-regulatory genes. The histone-DNA complex was immuno-precipitated with a specific H2A.Z antibody followed by qPCR. The results revealed that H2A.Z was incorporated at the promoters and TSS, but not the gene bodies, of cell-cycle-regulatory genes in wild type, but were reduced in *osarp6* mutants (Figure 5a,b). Therefore, our results confirmed the role of *OsARP6* in mediating H2A.Z deposition at cell-cycle-related genes.

## 4. Discussion

In this study, we characterized OsARP6 as conserved subunit of putative SWR1-C in rice. OsARP6 interacted with OsPIE1, a rice ortholog of yeast Swr1. We found that *OsARP6* is involved in vegetative and reproductive development of rice. Loss of *OsARP6* function resulted in pleiotropic defects in development including defect in height, tiller numbers, and fertility. The semi-dwarf phenotype was due to the defect in cell proliferation. The transcription levels and H2A.Z levels at promoters and TSS of *OsCDKA;1*, *OsCDKA;2*, *OsCYCB1;4*, *OsCYCB2;2 OsCYCA3;2* and *OsR2* were reduced in *osapr6* mutants.

ARP6 has been identified in yeast as a component of the SWR1-C [8,12]. Biochemical characterization of the yeast SWR1-C revealed that Arp6, Swc6, Swc2, and Swc3 form a sub-complex that associates to Swr1 [45]. Similarly, *Arabidopsis* ARP6 and SWC6 form a sub-complex that associates with PIE1 [18]. Phylogenetic analysis, interaction between OsARP6 and OsPIE1 and role in H2A.Z deposition confirmed that OsARP6 is a component of conserved SWR-C in rice.

The knockout *osarp6* mutants displayed pleiotropic developmental alterations including reduced plant height, reduced tiller number, attenuated panicle exertion and defects in reproductive development. In *Arabidopsis*, loss of function mutations in *PIE1*, *ARP6* or *SWC6* cause similar morphological and developmental phenotypic defects affecting vegetative and reproductive traits, including dwarf stature and early flowering and cause mis-expression of several genes [9,16,17,20,23,46,47]. Similarly, *Arabidopsis yaf9a yaf9b* double mutant plants and plants with reduced levels of *SWC4* expression (*swc4i*) display pleiotropic phenotypic alterations in both vegetative and reproductive development, including early senescence and chlorotic leaves, abnormal flowering and reduced plant and organ size [21,22]. Consistent with the role of SWR1-C in H2A.Z deposition, the *Arabidopsis H2A.Z* mutants also exhibit pleiotropic phenotypes [14]. Our results are consistent with previous results showing that putative rice SWR1-C and H2A.Z play key roles throughout plant development.

We found that reduced height of *osarp6* is due to reduced number of cells in the elongation zone, suggesting defect in cell division. Chromatin-remodeling has been reported to play role in transcriptional regulation during the cell cycle [48,49]. *Arabidopsis arp6* mutants have remarkably smaller leaves that are composed of fewer cells rather than a normal number of smaller cells [23]. Plants defective in other nuclear *ARPs*, such as *ARP4* and *ARP7*, also exhibit dramatic and remarkably diverse developmental phenotypes, including reduced cell size and/or numbers [19,50,51]. Consistent with previous studies, our results demonstrate that the reduced size of *osarp6* is due to defect in cell division.

In *Arabidopsis*, the presence of H2A.Z in the +1 nucleosome of certain genes is required for maintenance of transcriptional activity [17,52,53]. Our results indicate that OsARP6 regulate cell division by modulating H2A.Z deposition at the +1 nucleosome and not the gene bodies of cell-cycle-regulatory genes. Together, our results suggest that OsARP6 may contribute to the regulation of gene expression through modulation of H2A.Z dynamics.

## Figures and Tables

**Table 1:** Segregation analysis of heterozygous *osarp6* self-pollinated plants.

Parents (Self-Pollinated)	Offsprings					
	Total	WT	Heterozygous	Homozygous	Observed Ratio	Expected Ratio
*osarp6-1* (Heterozygous)	119	32	60	27	1:1.88:0.84	1:2:1
*osarp6-2* (Heterozygous)	134	37	68	29	1:1.83:0.78	1:2:1
Total	253	69	128	56	1:1.85:0.81	1:2:1

Chi-square test was used to evaluate the data, *X*^2^ = 1.37 and *P* > 0.05.

**Table 2:** Internode and panicle length analysis of wild-type and *osarp6* plants.

Genotype	Internode Length (cm)					Panicle Length (cm)
	Fifth	Fourth	Third	Second	First	
WT	32.2 ± 1.8	15.7 ± 1.3	9.3 ± 1.2	3.3 ± 1.4	1.1 ± 0.4	18.6 ± 1.2
*osarp6-1*	14.7 ± 0.6	5.0 ± 1.0	1.2 ± 0.2	0.8 ± 0.1	0.4 ± 0.1	14.7 ± 1.5
*osarp6-2*	14.5 ± 0.5	4.6 ± 0.5	1.3 ± 0.1	0.7 ± 0.1	0.4 ± 0.0	14.2 ± 0.6
*osarp6-1* (heterozygous)	24.7 ± 0.5	10.6 ± 1.5	5.1 ± 0.2	3.1 ± 0.4	0.6 ± 0.1	15.4 ± 0.2
*osarp6-2* (heterozygous)	25.0 ± 0.7	10.0 ± 0.4	5.2 ± 0.5	3.1 ± 0.1	0.5 ± 0.1	15.4 ± 0.5

## Data Availability

The data presented in this study are available on request from the corresponding authors. Sequence data from this article can be found in the GenBank/EMBL data libraries under the following accession numbers: *OsARP6* (Os01g16414) and *OsPIE1* (Os02g0689800).

## Supplementary Materials

The following are available online at https://www.mdpi.com/article/10.3390/biom11081100/s1, Figure S1: Alignment of ARP6 proteins from rice, *Arabidopsis* and yeast with conventional actins; Figure S2: Expression pattern of *OsARP6* obtained from ePlant Rice; Figure S3: Sequence comparisons of wild type, and knockout *osarp6* mutants; Figure S4: Phenotypes of heterozygous *osarp6* plants; Table S1: Quantification of cell length and cell number in 5th internodes of wild type and *osarp6* mutants; Table S2: Primers used in this study.
