# Supplementary material for: OsARP6 Is Involved in Internode Elongation by Regulating Cell-Cycle-Related Genes
Source: Biomolecules. 2021 Jul 26;11(8):1100. doi: 10.3390/biom11081100 (PMC8393719; doi:10.3390/biom11081100)
Supplement: Supplementary file 1 [file biomolecules-11-01100-s001.zip › biomolecules-1288815-supplementary.pdf]

Supplementary Materials

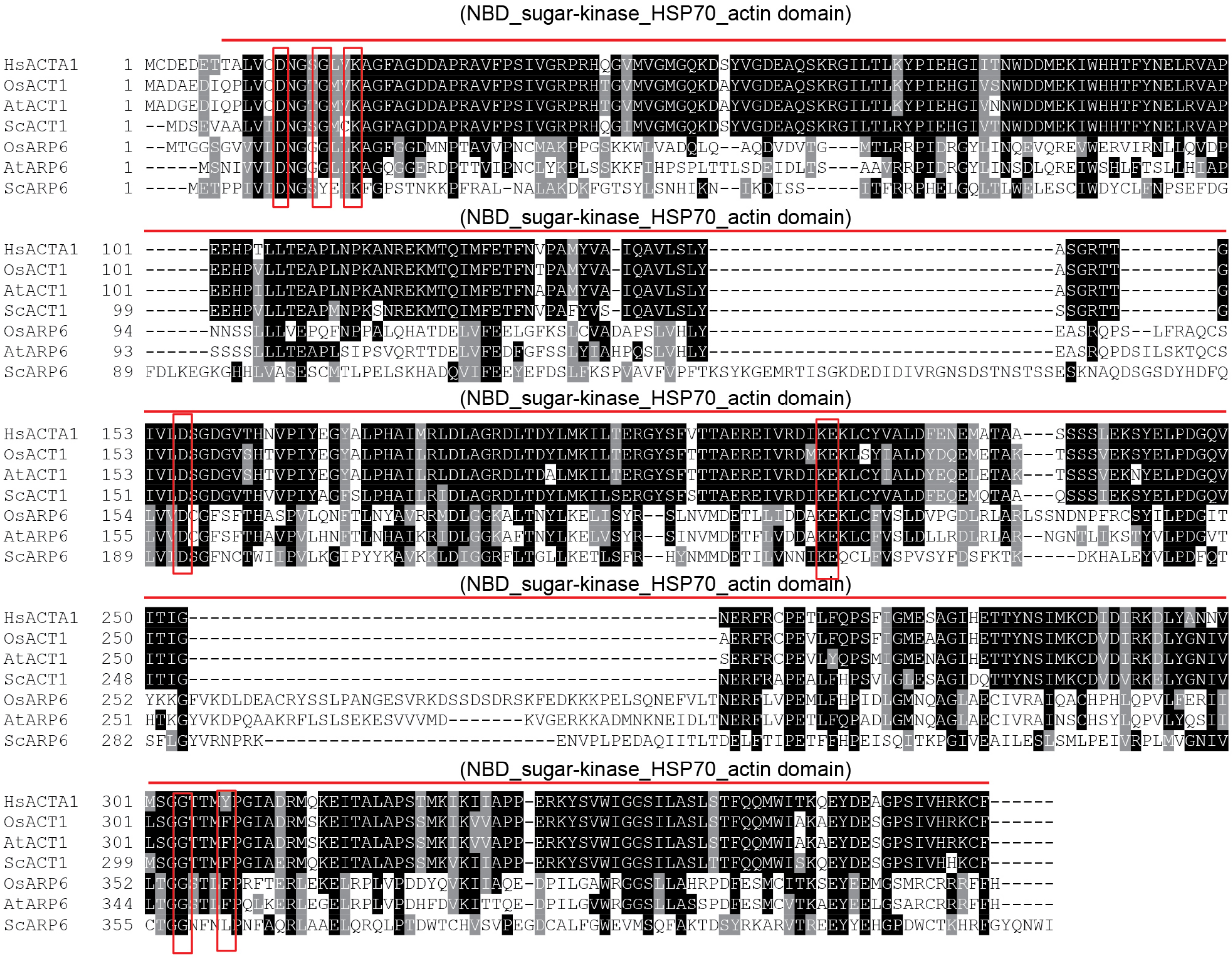

Figure S1. Alignment of ARP6 proteins from rice, *Arabidopsis* and yeast with conventional actins. Conventional actins from human, rice, *Arabidopsis* and yeast were used as reference. The Nucleotide-Binding Domain of the sugar ki-nase/HSP70/Actin super family (NBD_sugar-kinase_HSP70_actin) is shown with red line. The conserved amino acid residues involved in nucleotide binding are shown in red boxes. The sequences were aligned with MultAlin and visualized with BoxShade version 3.21.

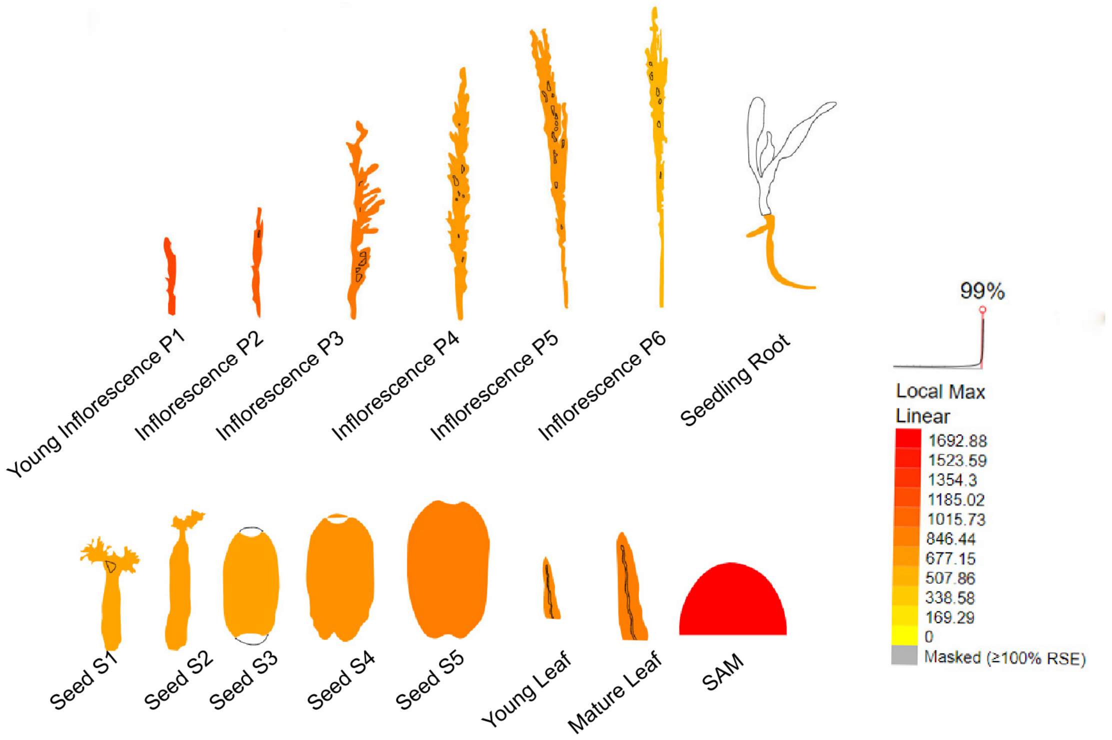

Figure S2. Expression pattern of OsARP6 obtained from ePlant Rice (http://bar.utoronto.ca/eplant_rice/). Expression profile showed that OsARP6 was highly expressed mainly in somatic apical meristem (SAM), young panicle, young leaf and mature leaf. The expression in root, mature panicle and early stages of seed development was low.

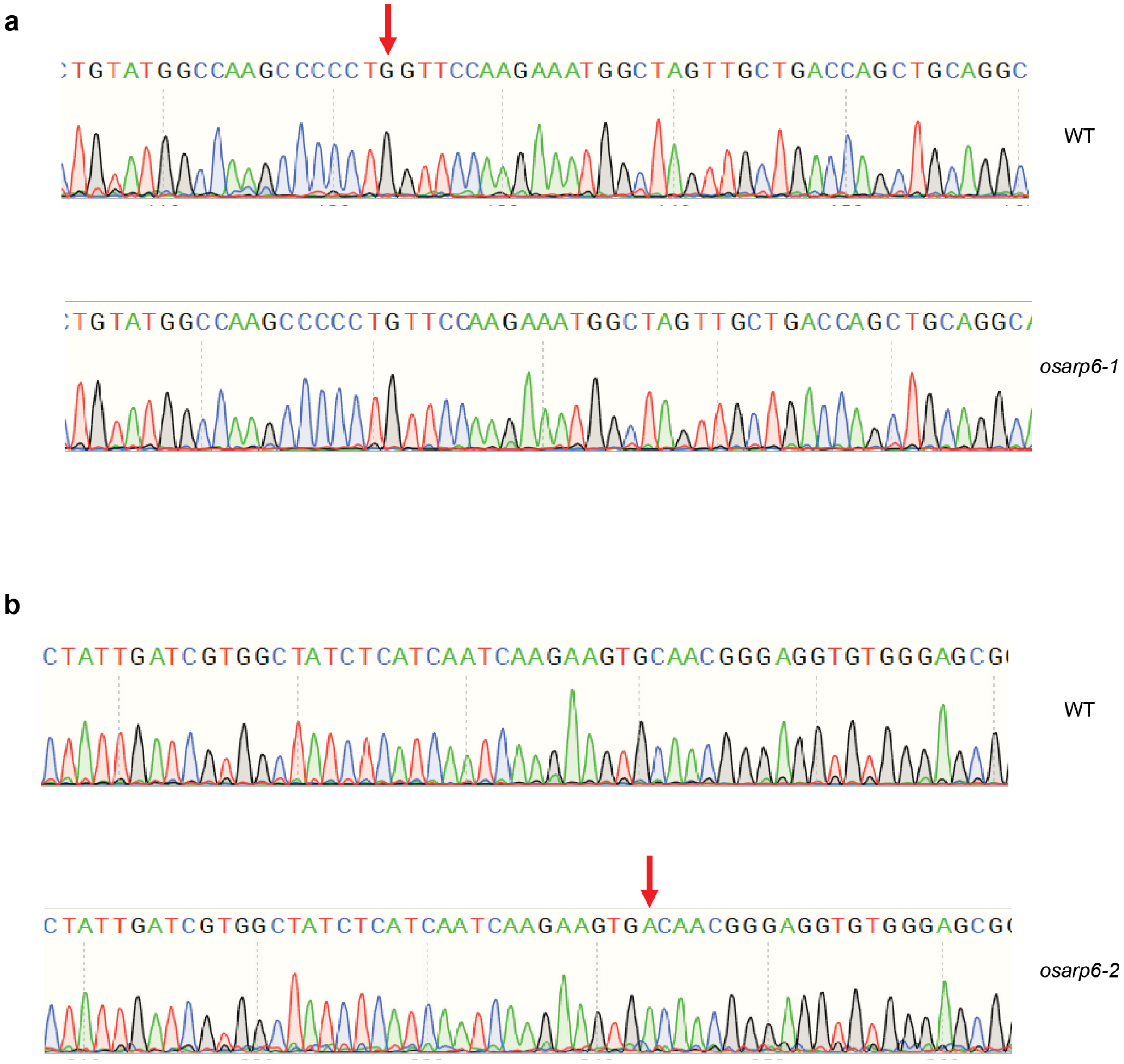

Figure S3. Sequence comparisons of wild type, and knockout *osarp6* mutants. (**a**) Sequence comparisons of wild type with *osarp6-1*. The deletion is marked with red arrow in wild type. (**b**) Sequence comparisons of wild type with *osarp6-2*. The insertion is marked with red arrow in *osarp6-2*.

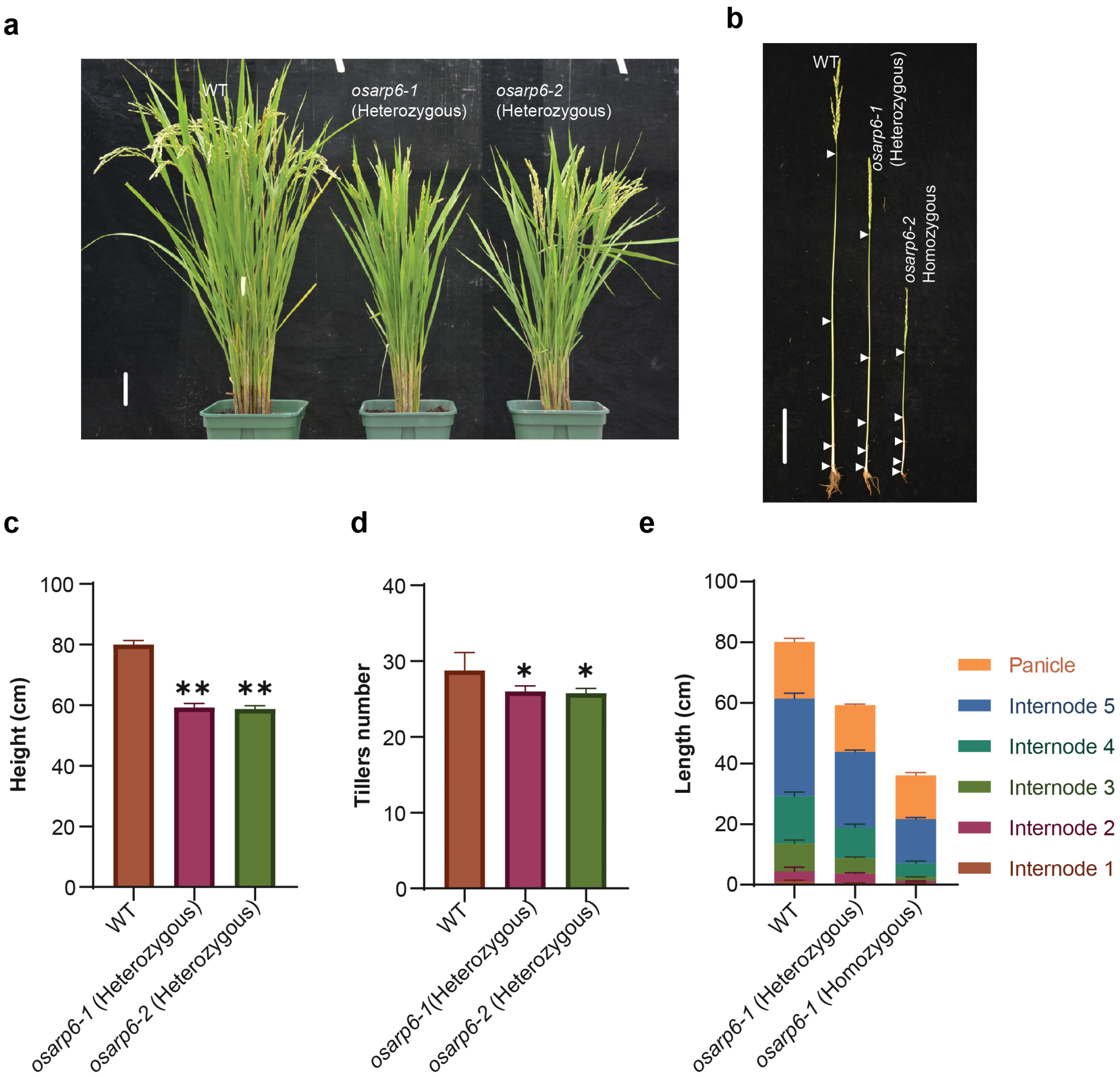

Figure S4. Phenotypes of heterozygous *osarp6* plants. (**a**) Representative image of 120-day-old wild type and *osarp6* (heterozygous) plants. Scale bar = 10 cm. (**b**) Culms of wild type and *osarp6-1* (heterozygous) and *osarp6* homozygous plants at mature stage. All leaves were removed to clearly show the elongated stems. White arrowheads indicate the nodes. Scale bar=10 cm. (**c**) Quantification of height of wild type and *osarp6-1* (heterozygous) plants. Error bars indicate SD (n=10). Asterisks indicate P <0.01(**) as determined by Student's *t* test analysis. (**d**) Quantification of tillers number from wild type and *osarp6-1* (heterozygous) plants. Error bars indicate SD (n=10). Asterisks indicate P <0.05 (*) as determined by Student's *t* test analysis. (**e**) Internodes and panicle lengths of wild type, *osarp6-1* (heterozygous) and *osarp6-1* (homozygous) plants at the mature stage. Error bars indicate SD (n=12).

Table S1. Quantification of cell length and cell number in 5th internodes of wild type and *osarp6* mutants

		Fifth internode length (cm)	Cell length (μm)	Cell number in a row /cm*	Estimated total cell number in a row in 5th internode
	WT	32.2 ±1.8	46.1±7.9	218.9±7.5	7.05x10³±13.5
	osarp6-1	14.7 ±0.6	46.9±8.1	212.7±4.6	3.13 x10³±2.76
	osarp6-2	14.5 ±0.5	45.7±8.6	221.3±8.8	3.21 x10³±4.4
Ratios	*WT/ osarp6-1*	2.19	0.98	1.03	2.25
	WT/ osarp6-1	2.22	1.01	0.99	2.20

*For quantification of cell number, three sections of 1 cm lengths were obtained from top, middle and bottom portions of the 5th internodes and the cell number in row was counted. The average data of the 9 sections for each genotype is shown.

Table S2. Primers used in this study

Cas9 targets

osarp6-1-sense

GGCAAGCCATTTCTTGGAACCAGG

osarp6-1-antisense

AAACCCTGGTTCCAAGAAATGGCT

osarp6-2-sense

GGCATCATCAATCAAGAAGTGCAA

osarp6-2-antisense

AAACTTGCACTTCTTGATTGATGA

Genotyping primers

osarp6-1 and osarp6-2

Forward primer (5′-AGGATAGGCAAGAATAGGAGGAA-3′)

Reverse primer (5′-TGAAAGAGAAGCCACAGTCAACA-3′)

Primers used for BiFC

OsARP6

Forward primer (5′-GGACTAGTATGACGGGTGGATCAGGTGTTG-3′)

Reverse primer (5′-CGGGGTACCGTGAAAGAATCTACGACGGCAC-3′)

OsPIE1

Forward primer (5′-GGACTAGTATGGAATTTTCATCAAGCCT-3′)

Reverse primer (5′-CGGGGTACCTCCGATGTCTGTGAAATCTGTA-3′)

Primers used for yeast two hybrid assay (Y2H)

OsARP6

Forward primer (5′-CCGGAATTCATGACGGGTGGATCAGGTGTTGTGG-3′)

Reverse primer (5′-CCGGAATTCTCAGTGAAAGAATCTACGACGGCAC-3′)

OsPIE1

Forward primer (5′-GGGTTTCATATGATGGAATTTTCATCAAGCCT-3′)

Reverse primer (5′-ACGCGTCGACTCATCCGATGTCTGTGAAATCTGTA-3′)

qPCR primers

OsARP6

Forward primer (5′-ACTTCGTCCTCTTGTGCCTG-3′)
Reverse primer (5′-GTGCGCCAAAAGAGATCCAC-3′)
Forward primer (5′-GGTGGATCAGGTGTTGTGGT-3′)
Reverse primer (5′-GGCCATACAGTTGGGGACAA-3′)
Forward primer (5′-GGTGTGGGAGCGGGTTATAC-3′)
Reverse primer (5′-TCACCAGGGACATCAAGGGA-3′)

OsCDKA;1

Forward primer (5′-ATCACGGCAACATCGTCAGG-3′)
Reverse primer (5′-AGTAAGCAACGCCGCGGAGTA-3′)

OsCDKA;2

Forward primer (5′-ACCACCGCATAGTCAAATCGTT-3′)
Reverse primer (5′-ACCACAATGTCACCACCTCGTGA-3′)

OsR2

Forward primer (5′-TCTGCACCTCCACTTCGCTCA-3′)
Reverse primer (5′-TAGGTGGTGGCCTTGGAAGCT-3′)

OsCYCA3;2

Forward primer (5′-GACATTCCTCAGGCGGTTCA-3′)
Reverse primer (5′-CGGAGACAGCCGTAGTCAAG-3′)

OsCYCB1;4

Forward primer (5′-AGCTCATGCCAGAGACCCTA-3′)
Reverse primer (5′-GAAGTCGTTCACCTCTGGGG-3′)

OsCYC-B2;2

Forward primer (5′-GGAGGAGTGAGGGTTTGAGC-3′)
Reverse primer (5′-GCGAGCCAAGAATCGGTCTA-3′)

Primers for ChIP-qPCR

OsACTIN1

Forward primer (5′- TGCGACGTGGATATTAGGAA-3′)
Reverse primer (5′- AAGGCAGTGATCTCCTTGCT-3′)

OsCDKA;1

Region 1
Forward primer (5′-CTGAGATCAGGATAGCTCTAGCAC-3′)
Reverse primer (5′-TTCCGCTTGTGTGGTTTAGTAG-3′)
Region 2
Forward primer (5′-AAGGCGCAGTCAAAGAGAGTC-3′)
Reverse primer (5′-GAGGAGAGGTGGGCAAAAGC-3′)
Region 3
Forward primer (5′-GCGGCGTTGCTTACTGTC-3′)
Reverse primer (5′-ACCTCGTGAGTAAACGTGCG-3′)

OsCDKA;2

Region 1

Forward primer (5′-GTACAAAGGTTCCTCACGGGC-3′)

Reverse primer (5′-CGTAATGCCTCCACACGAACAC-3′)

Region 2

Forward primer (5′-ATATGAGGAGAGAGCGACAATGC-3′)

Reverse primer (5′-TGGGAGTGGATGGGTTGTGG-3′)

Region 3

Forward primer (5′-CGTTCCTCTACCAGATTCTCCGG-3′)

Reverse primer (5′-AAGTCCGCGAGCTTCAATGAG-3′)

OsCYCB1;4

Region 1

Forward primer (5′-CCATACCTACACCCAACGGC-3′)

Reverse primer (5′-CGTTGCCTTTTTTACGTGGAGC-3′)

Region 2

Forward primer (5′-CTAGCTCGCTATATCTCGCCCC-3′)

Reverse primer (5′-CCTCCTCCTCTTGCCCAATC-3′)

Region 3

Forward primer (5′-ATTACATCGACACCCAGGTGGA-3′)

Reverse primer (5′-CATCGAGAGGTACCGGTCGA-3′)

CYC-B2;2

Region 1

Forward primer (5′-GTGCGGGTAGCCGTTAAGG-3′)

Reverse primer (5′-GGTAGGAAGGCGTAATTTGGCC-3′)

Region 2

Forward primer (5′-CCTTTGTCTCCCCTCTCCTCC-3′)

Reverse primer (5′-CCACCTCTCTCCCCCTCTC-3′)

Region 3

Forward primer (5′-ACATTGAGGAGGCAGCACC-3′)

Reverse primer (5′-CCTCACTCCTCCTGTAGAAGCTG-3′)

OsCYCA3;2

Region 1

Forward primer (5′-AATTCCATGGAATTCCCAATGCC-3′)

Reverse primer (5′-CATGACTCGGCGCCAAAAC-3′)

Region 2

Forward primer (5′-GCCCGCGATCCCCAGATTC-3′)

Reverse primer (5′-CGCGCCTACGAGATGAATCG-3′)

Region 3

Forward primer (5′-GAGATTAGCCCTCCTCATGTGG-3′)

Reverse primer (5′-GATTGCCCATCTCGAACTCGAG-3′)

OsR2

Region 1

Forward primer (5′-CCTGGTTTTTCTGAAGGAGGTCC-3′)

Reverse primer (5′-TCCCGACCGTTCGAGTTCG-3′)

Region 2
Forward primer (5′-AAGCGCACACAAGGACCTC-3′)
Reverse primer (5′-GAGGCGAGAGAGGGTGACG-3′)
Region 3
Forward primer (5′-AGGTCTTTGCACGATGGTACC-3′)
Reverse primer (5′-CTGGAGAAACGGTCGCCTAAG-3′)
